# Obesity, Type 2 Diabetes, and the Risk of Carpal Tunnel Syndrome: A Two-Sample Mendelian Randomization Study

**DOI:** 10.3389/fgene.2021.688849

**Published:** 2021-07-22

**Authors:** Jiarui Mi, Zhengye Liu

**Affiliations:** ^1^Master’s Programme in Biomedicine, Karolinska Institutet, Stockholm, Sweden; ^2^Department of Orthopedics, Zhongnan Hospital of Wuhan University, Wuhan, China

**Keywords:** Mendelian randomization, obesity, type 2 diabetes, carpal tunnel syndrome, body mass index

## Abstract

Some previous observational studies have reported an increased risk of carpal tunnel syndrome (CTS) in patients with obesity or type 2 diabetes (T2D), which was however, not observed in some other studies. In this study we performed a two-sample Mendelian randomization to assess the causal effect of obesity, T2D on the risk of CTS. Single nucleotide polymorphisms associated with the body mass index (BMI) and T2D were extracted from genome-wide association studies. Summary-level results of CTS were available through FinnGen repository. Univariable Mendelian randomization (MR) with inverse-variance-weighted method indicated a positive correlation of BMI with CTS risk [odds ratio (OR) 1.66, 95% confidence interval (CI), 1.39–1.97]. Genetically proxied T2D also significantly increased the risk of CTS [OR 1.17, 95% CI (1.07–1.29)]. The causal effect of BMI and T2D on CTS remained consistent after adjusting for each other with multivariable MR. Our mediation analysis indicated that 34.4% of BMI’s effect of CTS was mediated by T2D. We also assessed the effects of several BMI and glycemic related traits on CTS. Waist circumference and arm fat-free mass were also causally associated with CTS. However, the associations disappeared after adjusting for the effect of BMI. Our findings indicate that obesity and T2D are independent risk factors of CTS.

## Introduction

Carpal tunnel syndrome (CTS) is an entrapment neuropathy caused by the compression of the median nerve in the carpal tunnel of the waist. CTS is a common cause of work disability, with an incidence rate of around 1–5% in the general population, which incurs a high healthcare cost to the society (Atroshi et al., 1999; Shiri et al., 2011). Despite the high incidence rate of CTS, the cause of most CTS cases is unknown (Atroshi et al., 1999; Sternbach, 1999). Some lines of evidence exist on the role of metabolic factors including BMI, type 2 diabetes (T2D), and lipid levels in CTS (Werner et al., 1994; Karpitskaya et al., 2002; Geoghegan et al., 2004; Bland, 2005b; Gulliford et al., 2006; Balci and Utku, 2007). However, the relationships still remain controversial, as the observations are not consistent in other studies (Frost et al., 1998; Shiri et al., 2011). Furthermore, these findings mainly come from cross-sectional studies or cohort studies, which may be affected by confounding factors or reverse causality.

Mendelian randomization (MR) is a method that uses genetic variants [usually single nucleotide polymorphisms (SNPs)] as instrumental variables of exposures (e.g., BMI or T2D) for estimating the causal associations between the exposures and certain outcomes (Davey Smith and Ebrahim, 2005). MR is conceptually similar to a randomized controlled study as genetic variants are assigned randomly during gametes formation, prior to the disturbance of any confounding factor (Lawlor et al., 2008; Davey Smith and Hemani, 2014). Furthermore, the alleles of different individuals are fixed and cannot be altered by the onset and progression of diseases (Davey Smith and Ebrahim, 2005; Burgess and Thompson, 2015). Thus, the causal inferences obtained from MR analyses are less prone to biases derived from residual confounders and reverse causality. Here, we conducted an MR study to assess the causal effects of BMI, T2D, and several BMI associated traits on the risk of CTS. Furthermore, we also performed multivariable MR, a newly developed extension of MR that estimates the causal effect of a risk factor on the outcome not mediated by the other risk factors by analyzing several risk factors simultaneously.

## Materials and Methods

### Study Design and Data Source

**FIGURE 1:**
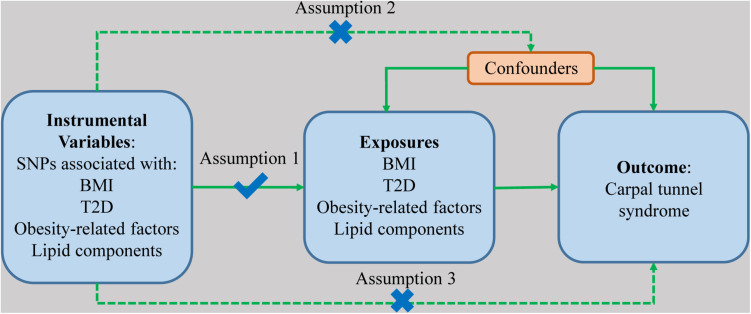
The diagram of the study design and three assumptions of Mendelian randomization. The obesity-related exposures include arm fat mass, arm fat-free mass, and waist circumference. Lipid components include HDL-cholesterol, LDL-cholesterol, total cholesterol, and triglycerides. SNP, single nucleotide polymorphism; BMI, body mass index; T2D, type 2 diabetes.

The summary of the study design is displayed in Figure 1, showing three major assumptions of MR study. First, the genetic variants are supposed to have a direct effect on the risk of the outcomes. Second, the genetic variants have no associations with any confounding factors. Lastly, the effects of the genetic variants on the risk of outcomes are only mediated by the exposures. Publicly available summary-level data of genetic instruments associated with BMI were extracted from published genome-wide association studies (GWASs) from Genetic Investigation of ANthropometric Traits (GIANT) consortium which explored genetic variants modulating human size and shape (Speliotes et al., 2010). Summary-level data of T2D was extracted from the largest T2D GWAS meta-analysis with 62,892 cases and 596,424 controls (Xue et al., 2018). Summary-level data of arm fat mass, arm fat-free mass, and waist circumference were obtained from UK BioBank.^1^ Associations of SNPs related to the exposures with CTS were obtained from FinnGen consortium (4505 cases and 86,854 controls).^2^

Firstly, we investigated the causal association between each exposure and the risk of CTS by using univariable MR. Next, we did multivariable MR analysis with each exposure adjusted for BMI. We also adjusted the effect of BMI on CTS for T2D. We also assessed the causal effect of several obesity-related factors including arm fat mass, arm fat-free mass, waist circumference, lipid profile, and glycemic traits on the risk of CTS with summary data from publicly available GWAS (Scott et al., 2012; Willer et al., 2013; Wheeler et al., 2017). Finally, we assessed the indirect effect of BMI on CTS *via* T2D and the indirect effect of T2D on CTS *via* BMI with the method as previously described (Burgess et al., 2015, 2017). Detailed information of the data sources is listed in Supplementary Table 1.

Genetic instrumental variables for the exposures were selected at a genome-wide significance level (*P* < 5 × 10^–8^). The population included in this study is refined in European ancestry. PLINK clumping method was used for calculating the linkage disequilibrium (LD) among the selected single nucleotide polymorphisms (SNPs). The criteria for LD were defined as SNPs with *R*^2^ > 0.01 and physical distance within 5000 kb. These SNPs were excluded from the subsequent analyses. We calculated mean *F*-statistic as instrumental strength with the method described by Bowden et al. to test for weak instruments (*F* < 10) (Supplementary Table 2; Bowden et al., 2016b).

### Statistical Analysis

Briefly, univariable MR analysis was firstly used to assess the causal associations of the selected exposures with CTS, respectively. In detail, inverse-variance-weighted (IVW) method was used as the main analysis for MR. We performed random effect IVW when ≥3 SNPs were included for the exposure and fixed effect IVW when only one or two SNPs were available. In addition, we performed weighted median method, MR-egger method, and MR-PRESSO (Mendelian Randomization Pleiotropy RESidual Sum and Outlier) method for sensitivity analyses. The weighted median method was also used to provide consistent estimates when up to 50% of the weight in the analysis were originated from invalid instrumental variables (Bowden et al., 2016a). The MR-Egger method can identify and correct potential pleiotropy (*P* for intercept <0.05) and gives a consistent estimate (Burgess and Thompson, 2017). We used the MR-PRESSO method to detect possible outliers in the analyses, and to obtain adjusted causal estimates after excluding the outliers. Lastly, in order to validate that the other causal effects are not mediated by BMI, we performed multivariable MR analysis to adjust the effects for BMI (Verbanck et al., 2018). Similarly, we also estimated the effect of BMI with adjustment for T2D on CTS. We subsequently calculated the proportion of BMI and T2D’s effects on CTS mediated by each other with MR-PRESSO adjusted coefficients (Burgess et al., 2015, 2017). All statistical analyses were two-sided and a *p*-value <0.0042 (0.05/12 adjusted with Bonferroni method) was considered significant for univariable MR, and a *p*-value between 0.0042 and 0.05 was considered significant. The analyses were conducted using R (version 4.0.2), TwoSampleMR (0.5.5), MR (0.5.5), and MR-PRESSO packages (Hemani et al., 2018; Verbanck et al., 2018; Sanderson et al., 2019).

## Results

### BMI and T2D on CTS

**FIGURE 2:**
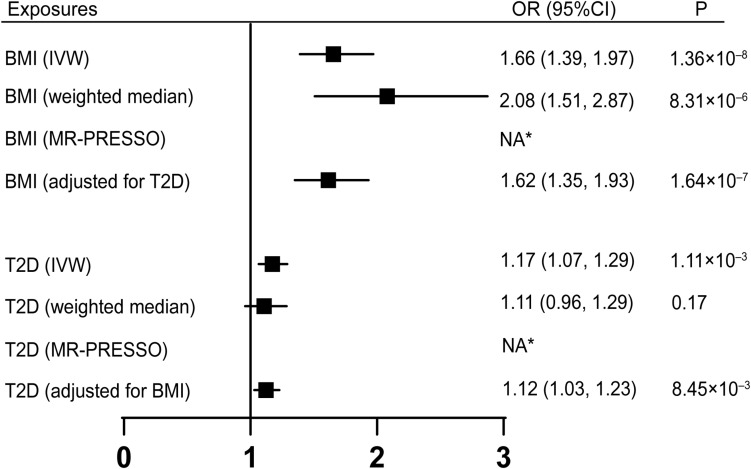
Univariable MR and multivariable MR analyses showed genetically proxied BMI and T2D were independent risk factors of carpal tunnel syndrome. Univariable MR was performed with IVW, weighted median, and MR-PRESSO methods. Multivariable MR was performed to assess the effect of T2D on CTS adjusted for BMI and the effect of BMI adjusted for T2D. *No outlier detected. IVW, inverse-variance-weighted; BMI, body mass index; T2D, type 2 diabetes; OR, odds ratio; CI, confidence interval; MR, Mendelian randomization.

Univariable MR was firstly performed for assessing the effects of BMI, T2D on the risk of CTS (Figure 2). Of note, for 1-SD increase in BMI level, the odds ratios (ORs) of CTS were 1.66 [95% confidence interval (CI), 1.39–1.97, *p* = 1.36 × 10^–8^] with IVW method, 2.08 (95% CI, 1.51–2.87, *p* = 8.31 × 10^–6^) with weighted median method. After adjusting for T2D, BMI was still causally associated with an increased risk of CTS, with an OR of 1.62 (95% CI, 1.35–1.93, *p* = 1.64 × 10^–7^). T2D was also revealed to be associated with an increased risk of CTS with an OR of 1.12 (95% CI, 1.07–1.29, *p* = 1.11 × 10^–3^) by using IVW method. After adjusting for BMI, the causal association of T2D with the risk CTS remained consistent (OR, 1.12, 95% CI, 1.03–1.23, *p* = 8.45 × 10^–3^), suggesting that T2D’s effect on CTS is independent of BMI. No horizontal pleiotropy was detected by MR-Egger in the above analyses. The direction and significance of these associations were maintained with MR-PRESSO analyses as no outlier was detected (Figure 2 and Supplementary Table 2).

Furthermore, we performed a mediation analysis to calculate the indirect effect of BMI on CTS *via* T2D and the indirect effect of T2D on CTS *via* BMI. The mediation effect of T2D in the causal pathway from BMI to CTS was 0.1735 (95% CI, 0.069–0.279, *p* = 1.18 × 10^–4^) with a mediation proportion of 34.4% (Table 1). Likewise, the mediation effect of BMI was −0.009 (95% CI, −0.016, −0.003) in T2D’s effect on CTS, with a mediation proportion of −5.7% (Table 1).

**TABLE 1:** Mediation effect of body mass index on carpal tunnel syndrome *via* type 2 diabetes and type 2 diabetes on carpal tunnel syndrome *via* body mass index.

**Exposure**	**Mediator**	**Total effect^a^**	**Effect X^b^**	**Effect M^c^**	**Mediation effect^d^**		**Mediated proportion**
	
		**Effect size (95% CI)**	**Effect size (95% CI)**	**Effect size (95% CI)**	**Effect size (95% CI)**	***p*-values**	**(%) (95% CI)**
BMI	T2D	0.505 (0.330, 0.679)	1.087 (1.034, 1.139)	0.160 (0.064, 0.256)	0.174 (0.069, 0.279)	1.18 × 10^–4^	34.4% (20.9, 41.0)
T2D	BMI	0.160 (0.064, 0.256)	−0.018 (−0.029, −0.006)	0.505 (0.330, 0.679)	−0.009 (−0.016, −0.003)	0.017	−5.4% (−25.6, −1.2)

*^*a*^Total effect: the effect of exposures on carpal tunnel syndrome.*

*^*b*^Effect X: the effect of exposures on mediators.*

*^*c*^Effect M: the effect of mediators on carpal tunnel syndrome.*

*^*d*^Mediation effect: the effect of exposures on carpal tunnel syndrome *via* mediators.*

### Obesity-Related Factors, Lipid Panel, and Glycemic Traits With CTS

**FIGURE 3:**
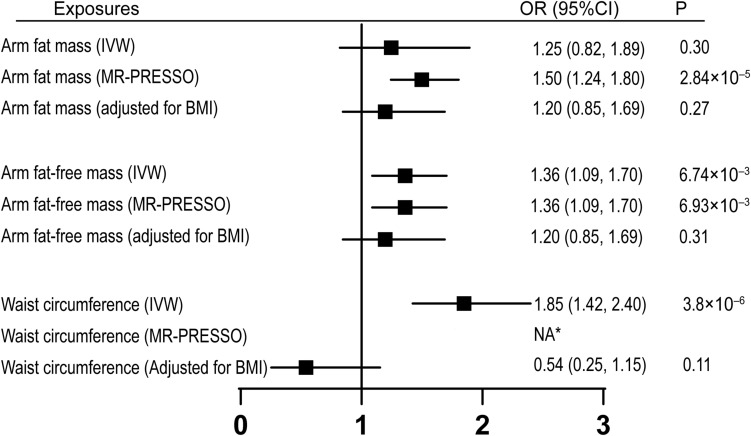
Univariable MR and multivariable MR analyses showing the associations of genetically proxied obesity-related factors with carpal tunnel syndrome. 1 SD increase of arm fat mass, arm fat-free mass, and waist circumference showed increased risk of carpal tunnel syndrome with outliers adjusted by MR-PRESSO. However, the causal associations were absent after adjustment for BMI. *No outlier detected. IVW, inverse-variance-weighted; BMI, body mass index; SD, standard deviation; OR, odds ratio; CI, confidence interval; MR, Mendelian randomization.

Next, we investigated if obesity-related factors (arm fat mass, arm fat-free mass, and waist circumference) and lipid components (HDL-cholesterol, LDL-cholesterol, total cholesterol, and triglycerides) are causally associated with CTS. Univariable MR analyses indicated that waist circumference and arm fat-free mass, but not arm fat mass, were causally associated with increased risk of CTS by using IVW methods (Figure 3). After adjusting for outliers with MR-PRESSO method, all the three traits were significantly correlated with CTS (Figure 3). Since these three factors are highly related to BMI, multivariable MR analyses with adjustment for BMI were subsequently performed. Our results showed that none of these factors remained significantly associated with CTS after adjusting for BMI, suggesting that the effects of these obesity-related measurements on CTS were mediated through BMI. In addition, we did not see any significant association between different lipid components and CTS, suggesting that dyslipidemia had no association with the incidence of CTS (Supplementary Table 2). We further assessed the effect of glycemic traits on the risk of CTS. While fasting insulin and fasting glucose levels were not causally associated with CTS, the association between HbA1C and CTS was significant (OR = 2.05, 95% CI, 1.01–4.15, *p* = 0.046).

## Discussion

Rates of overweight and obesity have been growing continuously in the past years, and have become major health problems worldwide. According to the World Health Organization (2016), 39% of adults over 18 years were overweight, and around 13% of the world’s adult population were obese. BMI is a commonly used index for overweight (BMI 25 –29.9) and obesity (BMI ≥30), defined as the weight in kilograms divided by the square of his height in meters (kg/m^2^). Overweight and obesity are found to be risk factors of a number of diseases, including CTS (Werner et al., 1994; Karpitskaya et al., 2002; Geoghegan et al., 2004; Bland, 2005b; Balci and Utku, 2007). However, due to the limitations of the previous studies, the conclusions may be affected by biases including confounders and reverse causality. In this study, we have assessed the causal association between BMI and CTS with MR. We found that genetically proxied BMI were positively correlated with the risk of CTS. The relationship remained consistent after adjusting for the effect of T2D, another metabolic risk factor for CTS (Figure 2; Karpitskaya et al., 2002; Gulliford et al., 2006). This is consistent with previous findings that BMI independently increased the risk of CTS, regardless of hyperglycemia (Becker et al., 2002; Rydberg et al., 2020). Our mediation analysis suggested that BMI’s effect on CTS is primarily a direct effect, though T2D does exert an indirect effect on the association between BMI and CTS.

The mechanisms of how higher BMI values increased the risk of CTS is still unclear. Some studies have proposed that in obese patients, excess adipose tissue within the carpal tunnel gradually narrows the tunnel and finally leads to higher intracarpal canal pressures, a well-established reason of CTS (Werner et al., 1994; Bland, 2005a). In our study, after adjusting for arm fat mass and fat-free mass, BMI was still significantly correlated with risk of CTS, which indicated that BMI might affect CTS through other factors (data not shown). According to pathological studies of CTS, microvascular circulation of the median nerve is impaired by the increased intracarpal pressure and ultimately leads to axon loss (Ettema et al., 2004; Bland, 2007). The increased pressure is also associated with stimulation and thickening of the subsynovial connective tissue (Ettema et al., 2004). Besides, obesity is often involved in metabolic syndromes which may render the patients more prone to develop peripheral neuropathy (Callaghan and Feldman, 2013).

Besides, we also assessed the association between several other obesity-related traits with the risk of CTS in this study including T2D, waist circumferences, arm fat mass/fat-free mass, cholesterols, and triglyceride levels. Among them, T2D, waist circumferences, arm fat mass, and arm fat-free mass were shown to be positively associated with the risk of CTS. However, after adjusting for the effect of BMI, only the association between T2D and CTS remained, indicating a causal effect of T2D on CTS which is independent of BMI. These findings are consistent with previous publications that the prevalence of T2D and increased BMI is higher in patients diagnosed of CTS (Karpitskaya et al., 2002; Rydberg et al., 2020). A meta-analysis including 36 studies (cross-sectional, case-control, and cohort studies) also suggested that T2D is a risk factor of CTS. The contribution of T2D to the risk of CTS was thought to be multifactorial, involving the effect of altered metabolism and vascular factors which renders the peripheral nerve more vulnerable to compression (Dahlin et al., 1986; Tapadia et al., 2010; Callaghan and Feldman, 2013; Rydberg et al., 2020). In patients with T2D, axonal degeneration and segmental demyelination due to chronic hyperglycemia, increased oxidative stress due to increased reactive oxygen species production in the mitochondria, pathological glycation of intracellular proteins, and also reduced microcirculation in peripheral nerves all contribute to the development of CTS (Tomlinson and Gardiner, 2008; Shimizu et al., 2011; Feldman et al., 2019). After open carpal tunnel release, patients with T2D reported worse outcomes and longer recovery time, particularly in patients with diabetic retinopathy, which is used as a proxy for diabetic neuropathy (Zimmerman et al., 2019). Additionally, histopathological studies showed that diabetic CTS patients had higher rates of synovial edema, vascular proliferation, and increased vascular wall thickness (Tekin et al., 2015).

Waist circumference has been observed to increase the risk of CTS in previous publications, and has been proposed as a body predictor of CTS (Plastino et al., 2011; Mondelli et al., 2014). In our univariable MR, we also observed a positive correlation of waist circumference with CTS with MR-PRESSO, however, this association may be mediated by the effect of BMI since no significant result was observed after adjusting for BMI. In a cross-sectional study including 6254 subjects, LDL cholesterol, triglycerides were found to be associated with CTS in subjects aged 30–44, but not in other age groups or in the total population (Shiri et al., 2011). This is consistent with our findings that cholesterol and triglyceride levels were not significantly correlated to the risk of CTS.

There are several strengths of the current study. To our knowledge, this is the first study that uses the MR methods to assess the effects of BMI and T2D on CTS. The major advantage of MR design was that by employing SNPs as instrumental variables of exposures, it reduced the biases such as confounding factors and reverse causality, and thus strengthened the causal estimates. Besides, the genetic variants used as instrumental variables were extracted from largest GWAS studies to date, and has shown good validity when used in previous MR studies. The used source of exposures and outcome are from two samples. Additionally, the population included in this study was confined to individuals of European ancestry in order to reduce population stratification bias. We have also performed several sensitivity analyses to ensure the consistency of the results and test for potential horizontal pleiotropy.

In the meantime, our study also has several limitations. Defining the population to European descendent individual also restricted the generalizability of the results to other populations. Besides, the numbers of SNPs used for each exposure is large (220–456 SNPs), which increased the risk that our results were biased by invalid SNPs. Nonetheless, the consistent observations from other sensitivity analyses verified our conclusions.

In summary, our study provided evidence that genetically predicted increase in BMI and T2D are independent risk factors of CTS. Reducing body weight and treating T2D should be considered as primary preventions of CTS.

## Data Availability Statement

Publicly available datasets were analyzed in this study. This data can be found here: the original contributions presented in the study are included in the article/Supplementary Material, further inquiries can be directed to the corresponding author.

## Author Contributions

JM and ZL conceptualized and designed the study, analyzed the data, and wrote the manuscript. Both authors have read and agreed to the published version of the manuscript.

## Conflict of Interest

The authors declare that the research was conducted in the absence of any commercial or financial relationships that could be construed as a potential conflict of interest.

## Footnotes

1 https://data.bris.ac.uk/data/dataset/pnoat8cxo0u52p6ynfaekeigi

2 https://www.finngen.fi/en
